# Gestational reactive hypoglycaemia and adverse pregnancy outcomes: a systematic review and meta-analysis

**DOI:** 10.1186/s12884-025-08016-x

**Published:** 2025-08-26

**Authors:** Muhammad Pradhiki Mahindra, Michelle Jie, Muhammad Pradhika Mapindra, Sana Rehman, Owen Vaughan, Sara Hillman, Anna L. David, Dimitrios Siassakos

**Affiliations:** 1 EGA Institute for Women’s Health, University College London, London, UK; 2 Department of Obstetrics and Gynaecology, St. George’s University Hospital, London, UK; 3 Wellcome/EPRSC Centre for Interventional and Surgical Sciences, London, UK

**Keywords:** Gestational diabetes mellitus, Gestational reactive hypoglycaemia, Low birth weight, Pregnancy outcomes, Small for gestational age.

## Abstract

**Background:**

Reactive hypoglycaemia is a condition where blood glucose drops after a glucose load, and may be associated with adverse pregnancy outcomes. This study aimed to determine the association between gestational reactive hypoglycaemia (GRH) and the risk of adverse pregnancy outcomes including those related to diabetes.

**Methods:**

We performed a systematic review and meta-analysis by searching 4 databases: Medline, Embase, Web of science, and Maternity & infant care database, from inception to 1 December 2023. The outcomes of interest were any reported adverse pregnancy outcomes including large for gestational age (LGA), macrosomia, small for gestational age (SGA), fetal growth restriction (FGR), low birth weight (LBW), caesarean delivery, neonatal intensive care unit (NICU) admission, neonatal hypoglycaemia, polyhydramnios, 5-min APGAR score < 7 and preterm delivery. Risk of bias assessment was performed with Newcastle Ottawa scale. Subgroup analysis was also performed.

**Results:**

From 14,746 records, 42 studies were selected for full-text assessment. Thirty studies reporting on 114,148 participants, including 18,878 women with GRH, fulfilled eligibility criteria. Pregnancies with observed GRH had higher risk of SGA (RR = 1.49, 95%CI = 1.33, 1.68), LBW (RR = 1.35, 95%CI = 1.13, 1.60), FGR (RR = 1.21, 95%CI = 1.05, 1.41), and NICU admission (RR = 1.23, 95%CI = 1.02, 1.49) compared to the euglycaemic group. At subgroup analyses, GRH diagnosed at postload glucose < 3 mmol/l was associated with an increased risk of NICU admission (RR = 3.39, 95%CI = 1.56, 7.34); and GRH limited to post glucose tolerance test (GTT) was associated with increased risk of polyhydramnios (RR = 1.93, 95%CI = 1.17, 3.20) and SGA (RR = 1.90, 95%CI = 1.01, 3.58).

**Conclusions:**

GRH is a condition not routinely diagnosed in pregnancy but associated with adverse fetal-neonatal outcomes as SGA, FGR, and NICU admission. At GTT, GRH is associated with the risk of polyhydramnios. More studies are still necessary to determine the threshold value for diagnosis of GRH and explore associations with other outcomes related to glucose dysmetabolism.

**Supplementary Information:**

The online version contains supplementary material available at 10.1186/s12884-025-08016-x.

## Background

As part of normal physiological metabolic adaptation in pregnancy, increased maternal insulin resistance, lipolysis, and hepatic gluconeogenesis alongside reduced skeletal muscle glucose uptake is aimed for maintaining maternal glucose availability for the fetus [1–3]. Maternal and placental secreted hormones, particularly human placental lactogen, glucagon, cortisol, oestrogen, and progesterone all act in parallel to reduce maternal tissue sensitivity to insulin. Meanwhile, plasma insulin is elevated in mid-late gestation to maintain normal glucose homeostasis [3, 4]. Abnormal glucose tolerance that is detected for the first time in mid-late gestation can be clinically manifested as hyperglycaemia, and then diagnosed as gestational diabetes mellitus (GDM) according to various sets of criteria [5–7].

Hypoglycaemia during pregnancy is another condition of abnormal glucose values that mostly occurs due to the side-effect of tight glycaemic control in pregnancy diabetes [8–10]. However, pregnancy hypoglycaemia may also occur in women who have never been diagnosed with pregnancy diabetes and this condition may be an undiagnosed entity of impaired glucose tolerance. After a glucose load, some women might also exhibit hypoglycaemia considered as gestational reactive hypoglycaemia (GRH) [11, 12]. A previous meta-analysis by Mitta showed that 1-hour low glucose value following a 50-grams glucose challenge test (GCT) was associated with abnormal fetal growth [13]. Although the mechanism is not clearly understood, the asynchronous insulin response in GRH indicates a sign of glucose dysmetabolism, as akin to some form of impaired glucose tolerance or diabetes during pregnancy [14]. However, it is also not known if GRH in response to a glucose load as part of testing represents similar fluctuations in response to meals rich in simple carbohydrates in daily life.

Optimal screening of abnormal glucose tolerance during pregnancy is a part of essential maternity care to prevent impaired glucose tolerance-associated perinatal complications and postnatal progression of future morbidities in mother and child [15, 16]. Diagnostic criteria using a glucose loading test have been internationally adopted and modified to assess any glucose tolerance and insulin function in pregnant women population [6, 7, 15]. The Hyperglycaemia and Adverse Pregnancy Outcomes **(**HAPO) study found that values of 2-hour oral glucose tolerance testing (OGTT) predict adverse pregnancy outcomes related to pregnancy hyperglycaemia in a dose-response fashion [17, 18]. Nonetheless, the current interpretation of glucose loading tests only reflects hyperglycaemia and does not consider hypoglycaemia following a glucose loading test [12]. There is no current consensus established regarding the standards of glucose level to diagnose GRH and our understanding of its association with adverse pregnancy outcomes is limited. The previous meta-analysis has shown that low glucose values following a 1-hour GCT are a potential predictor of LBW and SGA but it did not explore other possible pregnancy outcomes [13]. The objective of this study was to investigate the association between GRH after any glucose load and pregnancy outcomes, by meta-analysis of the published literature.

## Methods

This study was registered on PROSPERO (https://www.crd.york.ac.uk/prospero/), a global registry of systematic review, that was funded by National Institutes of Health (CRD42023355124). This review was written in concordance with the guideline of Meta-Analysis of Observational Studies in Epidemiology [19], and the Preferred Reporting Items for Systematic Review and Meta-Analysis protocol [20].

### Search strategy

Initial literature searching was conducted using the following databases: Medline, Embase, Web of Science, and Maternity & Infant Care Database (MIDIRS). To obtain additional papers not identified in the primary search, the snowball method was utilised by searching relevant cited articles within reference lists of included studies. The literature search included studies from inception to 1 December 2023, for case-control and cohort studies. The keywords for the search strategy were agreed with a medical librarian from the university faculty of population health science. The algorithm used for the search strategy was performed by combining MeSH and keyword terms in the databases. Detailed information on terms used in our search strategy for all databases is shown in Supplementary File 1.

### Study selection and eligibility criteria

We reviewed studies that reported low glucose values following glucose ingestion during a GCT and/or a GTT as the exposure, which we defined as GRH in our meta-analysis. The study design considered appropriate for this systematic review was observational (case-control and cohort) studies that used either GCT or GTT for GDM screening/diagnosis ≥ 24 weeks. Published full-text articles and conference abstracts in any language regardless of publication year were collected. The population of interest for this systematic review and meta-analysis study was singleton pregnant women with GRH and not diagnosed with GDM or pregestational diabetes. Pregnancy with euglycaemia was determined as the control group. The outcomes of interests evaluated in this review were based on the adverse pregnancy outcomes in relation to glucose loading test and associated pregnancy outcomes [13, 17, 21], including small for gestational age (SGA), large for gestational age (LGA), low birth weight (LBW), macrosomia, caesarean delivery, NICU admission, polyhydramnios, neonatal hypoglycaemia, neonatal hyperbilirubinaemia, low 5 min APGAR score (< 7), fetal growth restriction (FGR), polyhydramnios, shoulder dystocia, perinatal mortality, preeclampsia (PE), and postpartum hemorrhage. Studies with confirmed pregestational diabetes, pre-existing type 1/2 diabetes mellitus, or diagnosed GDM in GRH cohort were excluded. Studies with incomplete information or evaluating low fasting glucose value, as opposed to post-load plasma glucose, were also excluded. Studies that were high in risk of bias assessment were also excluded.

### Screening, data extraction, and risk of bias assessment

Article screening was independently completed by three review authors (MPMH, MJ, and MPMP) using Rayyan web platform for systematic reviews. Data extraction from eligible studies was independently performed by three review authors (MPMH, MJ, and MPMP). All studies extracted from each database were screened for their eligibility by title and abstract, according to the eligibility criteria. In cases where the title or abstract did not sufficiently indicate whether they met the inclusion criteria, the full-text was reviewed. Full-text articles for the studies were retrieved, reviewed, and analysed to see if they were suitable and relevant for qualitative analysis. We manually hand-searched reference lists for additional related studies. All studies that matched the selected eligibility criteria were extracted using a dedicated data sheet. We extracted relevant information from all studies meeting our eligibility criteria: first author, publication year, country of each study, the definition of exposure, method of giving exposure, sample size, outcome of interest, outcome definition (if any), and effect measure (number or proportion with and without outcomes). Any conflicting statements between reviewers were resolved by discussions with the senior reviewer.

### Quality assessment

Measurement bias at study level for our review analysis was investigated using the Newcastle-Ottawa Scale for cohort and case-control studies [22]. Bias assessment was independently done by three reviewers (MPMH, MJ, and MPMP). According to the Newcastle-Ottawa Scale, the quality assessment is based on 3 criteria: study selection of participants (4 stars), study comparability (2 stars), and outcome assessment (3 stars). We considered studies with 7–9 stars as good, studies with 2–6 stars as fair, and studies with 0–2 stars as poor quality. Studies with poor scores in the Newcastle-Ottawa Scale were categorised as high risk of bias. Disagreements between the reviewers on the risk of bias assessment were resolved through discussion between the reviewers, and a third senior reviewer (DS) was consulted in cases where they were unable to come to an agreed conclusion.

### Statistical analysis

Meta-analysis and forest plot generation were conducted with Review Manager 5.4 and statistical significance was considered for any two-sided *p*-value less than 0.05. From each study the number of those with and without pregnancy complications in the two groups (GRH and normal glucose tolerance) was uniformly measured as a risk ratio (RR). Continuous data was collected and measured as standardized mean differences (SMD). A random-effect model and the Mantel-Haenszel method were used because of the high clinical heterogeneity across studies; each study used different criteria to define hypoglycaemia. I^2^ statistic was used to determine statistical heterogeneity. Data with I^2^ less than 40% were analysed using a fixed-effect model. Forest plots were employed to show the pooled estimates. To evaluate clinical heterogeneity, subgroup analysis explored different thresholds for GRH : < 5 mmol/l (90 mg/dl); < 4 mmol/l (70 mg/dl); and < 3 mmol/l (60 mg/dl). A further subgroup analysis evaluated the clinical manifestations of GRH, based on low value following either 1-hour GCT or GTT.

## Results

### Literature search

**Fig. 1:**
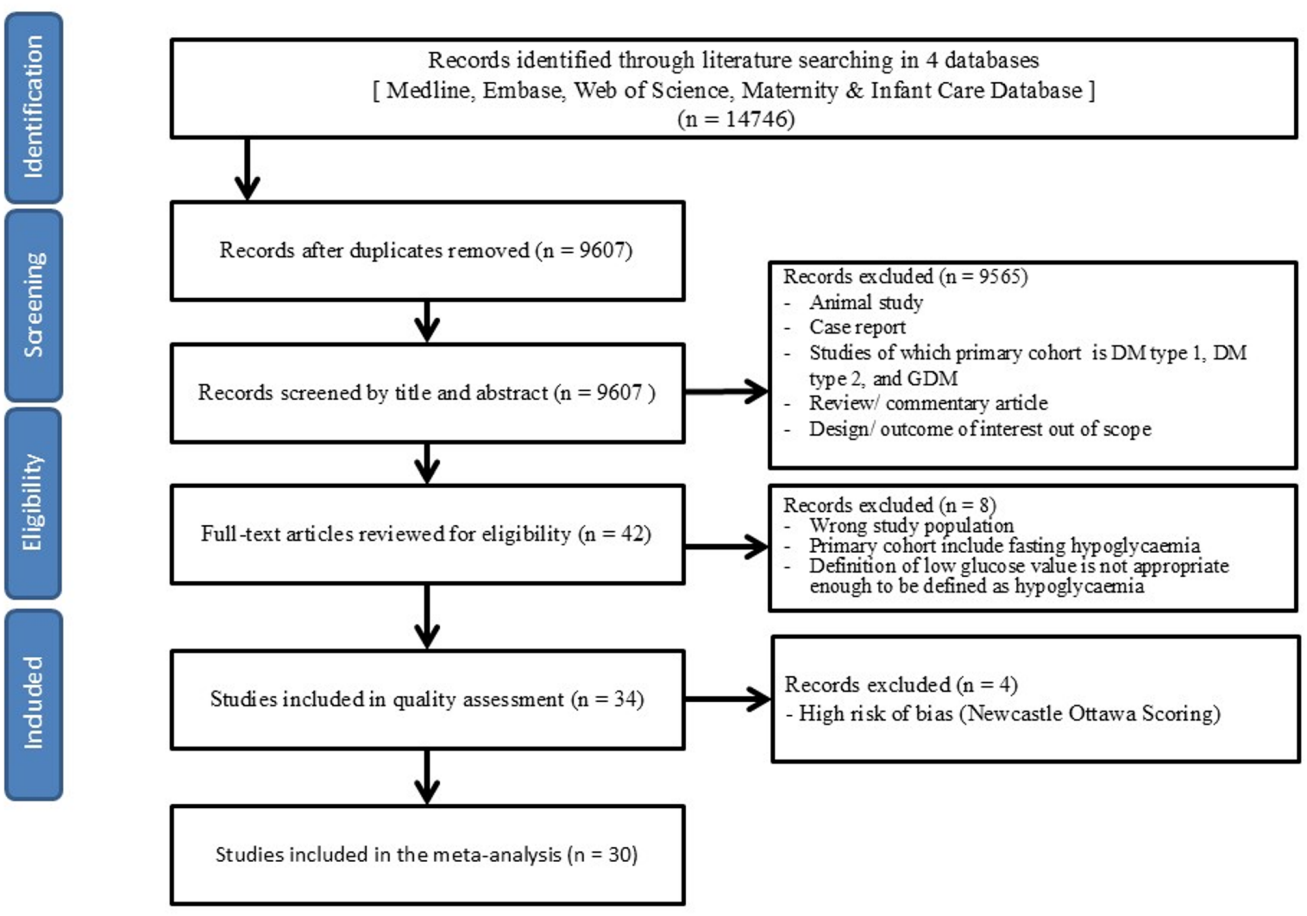
Flow chart of study inclusion in systematic review and meta-analysis

The PRISMA flow chart summarises the process to determine inclusion/exclusion for records and studies extracted from databases (Fig. 1). From the search strategy, 9607 of 14,746 records were yielded from all databases for the title and abstract screening, after removing duplicates. Throughout abstract screening, 42 full-text papers were obtained for eligibility assessment. After excluding 8 studies, 34 were included for the quality assessment, consisting of 29 full-text articles and 4 conference abstracts [23–26]. Based on the scoring under the Newcastle-Ottawa scale, three reviewers (MPMH, MPMP, and MJ) and the senior author excluded 4 studies [23, 27–29] with a high risk of bias for quantitative analyses (Table 1.). Reviewers’ decision on the bias assessment can be seen in Supplementary File 2. Overall, the quantitative analyses included 30 studies with a total of 114,148 participants, comprising 18,878 women with GRH and 95,270 euglycaemic control women.

**Table 1:** Quality assessment for eligible studies

Author, Year	Representativeness of the Exposed Cohort	Selection of the Non-Exposed Cohort	Ascertainment of Exposure	Outcome of Interest Was Not Present at Start of Study	Comparability of Cohorts on the Basis of the Design or Analysis	Assessment of Outcome	Follow-up Long Enough for Outcomes to Occur	Adequacy of Follow-up of Cohorts	Total Score			
									Selection Domain	Comparability Domain	Outcome/Exposure Domain	Quality
Bayraktar, 2020 [30]	*	*	*	*	**	*	*	*	4	2	3	Good
Bhat, 2012 [31]	*	*	no star	*	**	*	*	*	3	2	2	Good
Bienstock, 2008 [27]	no star	*	*	*	no star	*	*	*	3	0	3	Poor
Budak, 2018 [32]	*	*	*	*	**	*	*	*	4	2	3	Good
Calfee, 1999 [33]	*	*	no star	*	**	*	*	*	3	2	3	Good
Delibas, 2018 [34]	*	*	*	*	**	*	*	*	4	2	3	Good
Ding, 2023 [35]	*	*	*	*	**	*	*	*	4	2	3	Good
Duhl, 2000 [36]	*	*	*	*	*	*	*	*	4	1	3	Good
Feinberg, 2005 [37]	*	*	*	*	**	*	*	*	4	2	3	Good
Kerenyi, 2009 [28]	*	*	*	*	no star	*	*	no star	4	0	2	Poor
Kwon, 2015 [23]	no star	no star	no star	*	*	no star	*	*	1	1	2	Poor
Kwon, 2018 [38]	*	*	*	*	**	*	*	*	4	2	3	Good
Lurie, 1998 ± [39]	no star	*	no star	*	*	*	*	*	2	1	3	Fair
Ma, 1998 [40]	*	*	*	*	**	*	*	*	4	2	3	Good
Melamed, 2013 [41]	*	*	*	no star	**	no star	*	*	3	2	2	Fair
Nayak, 2019 [42]	*	*	*	*	*	*	*	no star	4	1	2	Good
Oawada, 2019 [43]	*	*	*	*	**	*	*	*	4	2	3	Good
Ong, 2008 [44]	no star	*	*	*	*	*	*	*	3	1	3	Good
Pugh, 2009 [45]	*	*	no star	*	*	*	*	*	3	1	3	Good
Raviv, 2021 [46]	*	*	*	*	*	*	*	no star	4	1	2	Good
Rehman, 2022 [47]	*	*	*	*	*	*	*	*	4	1	3	Good
Reicher, 2021 [48]	*	*	*	*	*	*	*	*	4	1	3	Good
Rottenstreich, 2017 ± [49]	no star	*	*	*	*	*	*	*	3	1	3	Good
Scholl, 2021 [29]	no star	*	*	*	no star	*	*	*	3	0	3	Poor
Shinohara, 2015 [50]	*	*	*	*	*	*	*	no star	4	1	2	Good
Shinohara, 2016 [51]	*	*	*	*	*	*	*	no star	4	1	2	Good
Stivers, 2020 [26]	*	*	*	*	*	*	*	*	4	1	3	Good
Tanacan, 2020 [52]	*	*	*	*	*	*	*	*	4	1	3	Good
TopÇu, 2016 [53]	*	no star	*	*	*	*	*	no star	3	1	2	Good
Vadakekut, 2010 [54]	*	*	*	*	*	*	*	*	4	1	3	Good
Vemareddy, 2009 [25]	*	*	*	*	**	*	*	*	4	2	3	Good
Yoles, 2021 [24]	*	*	no star	*	*	no star	*	*	3	1	2	Good
Yuen, 2019 [55]	*	no star	*	*	*	*	*	no star	3	1	2	Good
Weissman, 2005 [56]	*	*	*	*	*	*	*	*	4	1	3	Good

* >star symbol > fulfilment of eligibility criteria

± symbol > disagreement between reviewers, resolved by the senior author

### Study characteristics

Basic characteristics of 30 studies are summarized in Table 2. Most participants in the cohorts were recruited from the general pregnant population, except one article which included participants based on prior history of bariatric surgery [57]. The majority of studies performed 1-hour plasma glucose measurement after 50 g GCT. Nine studies performed GTT measurement [30, 57–64].

**Table 2:** Characteristics of the included studies in the systematic review and Meta-Analysis

Author, Year	Study Design; Setting	Enrolment	Gestational Reactive Hypoglycaemia Definition		Euglycaemia Definition	Pregnancy Outcomes
			Glucose Loading Test	Threshold Value		
Bayraktar, 2020 [30]	Retrospective Cohort; Turkey	24–28 weeks	Glucose tolerance test	≤ 3.9 mmol/l	3.9–8.5 mmol/l	LBW, caesarean delivery, NICU admission, preterm birth, macrosomia, APGAR score < 7
Bhat, 2012 [31]	Prospective Cohort; India	24–28 weeks	1-hour Glucose challenge test	≤ 4.9 mmol/l	5-7.8 mmol/l	LBW, NICU admission
Budak, 2018 [32]	Retrospective Cohort; Turkey	24–28 weeks	1-hour Glucose challenge test	< 5 mg/dL	5-7.8 mmol/l	SGA, caesarean delivery, 5-minute APGAR score < 7, preterm birth
Calfee, 1999 [33]	Prospective Cohort; USA	≥ 24 weeks	1-hour Glucose challenge test	≤ 4.9 mg/dL	5-7.8 mmol/l	FGR
Delibas, 2018 [34]	Retrospective Cohort; Turkey	25–28 weeks	Glucose tolerance test	< 2.5 mmol/l	Carpenter and Coustan thresholds	SGA, LGA, 5-minute APGAR score < 7, NICU admission, preterm birth, PE
Ding, 2023 [35]	Retrospective Cohort; USA	≥ 24 weeks	1-hour Glucose challenge test	< 4.6 mmol/l	≥ 4.6 mmol/l	SGA, NICU admission, neonatal hypoglycaemia, neonatal hyperbilirubinaemia, perinatal death, PE, postpartum hemorrhage
Duhl, 2000 [36]	Retrospective Cohort; USA	26–29 weeks	1-hour Glucose challenge test	PG < 4 mmol/l	5-7.1 mmol/l	SGA
Feinberg, 2005 [37]	Retrospective Case Control Study; USA	24–28 weeks	1-hour Glucose challenge test	< 4.9 mmol/l	4.9–7.8 mmol/l	LBW, caesarean delivery, 5-minute APGAR score < 7, NICU admission, preterm birth, neonatal hypoglycaemia, neonatal hyperbilirubinaemia, postpartum hemorrhage
Kwon, 2018 [38]	Retrospective Cohort; Korea	24–28 weeks	1-hour Glucose challenge test	< 4.8 mmol/l	4.8–7.2 mmol/l	SGA, LGA, LBW, macrosomia, caesarean delivery, 5-minute APGAR score < 7, NICU admission, preterm birth, polyhydramnios, PE, perinatal mortality
Lurie, 1998 [39]	Retrospective Cohort; Israel	24–28 weeks	1-hour Glucose challenge test	< 3.3 mmol/l	3.3–6.1 mmol/l	Macrosomia, cesarean delivery, polyhdramnios, neonatal hyperbilirubinaemia, PE
Ma, 1998 [40]	Retrospective Cohort; USA	24–30 weeks	1-hour Glucose challenge test	< 5mmol/l	5-6.7 mmol/l	SGA, LGA, cesarean delivery, NICU admission, neonatal hypoglycaemia, neonatal hyperbilirubinaemia, PE
Melamed, 2013 [41]	Retrospective Cohort; Israel	24–28 weels	1-hour Glucose challenge test	< 3.9 mmol/l	3.9–6.7 mmol/l	SGA, LGA, LBW, macrosomia, FGR, caesarean delivery, 5-minute APGAR score < 7, NICU admission, preterm birth, neonatal hypoglycaemia, neonatal hyperbilirubinaemia, shoulder dystocia, perinatal mortality, PE, postpartum hemorrhage
Nayak, 2019 [42]	Retrospective Cohort; UK	24–28 weeks	Glucose tolerance test	< 3.6 mmol/l	3.6–7.7 mmol/l	LBW
Oawada, 2019 [43]	Retrospective Case Control Study; Japan	24–28 weeks	1-hour Glucose challenge test	< 4.2 mmol/l	4.2–7.8 mmol/l	SGA, LGA, LBW, macrosomia
Ong, 2008 [44]	Retrospective Cohort; UK	27–29 weeks	1-hour Glucose challenge test	< 4 mmol/l	4.1–7.8 mmol/l	-
Pugh, 2009 [45]	Prospective Cohort; USA	24–28 weeks	1-hour Glucose challenge test	< 4.9 mmol/l	4.9–7.8 mmol/l	LBW, macrosomia, FGR, caesarean delivery, 5-minute APGAR score < 7, NICU admission, preterm birth, PE
Raviv, 2021 [46]	Retrospective Cohort; Israel	24–28 weeks	Glucose tolerance test	< 3.3 mmol/l	Carpenter and Coustan thresholds	SGA, LGA, LBW, macrosomia, shoulder dystocia, PE
Rehman, 2022 [47]	Retrospective Cohort; UK	24–28 weeks	Glucose tolerance test	< fasting glucose value	Euglycaemia within all values (FPG < 95 mg/dL, 1-hour PG < 180 mg/dL, 2-hour PG < 140 mg/dL on modified IADPSG criteria	SGA, polyhydramions, shoulder dystocia, perinatal mortality
Reicher, 2021 [48]	Retrospective Cohort; Israel	24–28 weeks	Glucose tolerance test	< 3.3 mmol/l	Carpenter and Coustan thresholds	LGA, CS, 5-minute APGAR score < 7, polyhydramnios, PE
Rottenstreich, 2018 [49]	Retrospective Cohort; Israel	24–28 weeks	Glucose tolerance test	< 3.1 mmol/l	> 3.2 mmol/l	SGA, LGA, LBW, macrosomia, caesarean delivery, preterm birth,
Shinohara, 2015 [50]	Retrospective Cohort; Japan	24–28 weeks	1-hour Glucose challenge test	< 5 mmol/l	5-7.8 mmol/l	SGA, preterm birth, caesarean delivery
Shinohara, 2016 [51]	Retrospective Cohort; Japan	24–28 weeks	1-hour Glucose challenge test	< 5 mmol/l	5-7.8 mmol/l	SGA, caesarean delivery
Stivers, 2020 [26]	Retrospective Cohort; USA	24–28 weeks	1-hour Glucose challenge test	< 4.9 mmol/l	4.9–7.2 mmol/l	SGA, FGR, LGA
Tanacan, 2020 [52]	Retrospective Cohort; Turkey	24–28 weeks	1-hour Glucose challenge test	< 4.1 mmol/l	4.1–7.8 mmol/l	SGA
TopÇu, 2016 [53]	Retrospective Case Control Study; Turkey	24–28 weeks	1-hour Glucose challenge test	< 4.9 mmol/l	4.9–7.2 mmol/l	SGA, LGA, LBW, macrosomia, caesarean delivery, NICU admission, preterm birth, polyhydramnios, PE, perinatal mortality
Vadakekut, 2010 [54]	Retrospective Cohort; USA	24–28 weeks	1-hour Glucose challenge test	< 4.9 mmol/l	4.9–7.5 mmol/l	SGA
Vemareddy, 2009 [25]	Retrospective Cohort; USA	24–28 weeks	1-hour Glucose challenge test	< 5 mmol/l	5-7.2 mmol/l	-
Yoles, 2021 [24]	Retrospective Cohort; Netherlands	No information	1-hour Glucose challenge test	< 5 mmol/l	5-7.8 mmol/l	preterm delivery, LBW, macrosomia
Yuen, 2019 [55]	Prospective Cohort; Australia	24–28 weeks	Glucose tolerance test	< 3.6 mmol/l	Carpenter and Coustan thresholds	caesarean delivery, NICU admission, neonatal hypoglycaemia, perinatal mortality
Weissman, 2005 [56]	Retrospective Cohort; Israel	24–28 weeks	Glucose tolerance test	< 2.8 mmol/l	2.8–7.2 mmol/l	SGA, LGA, macrosomia, cesarean delivery

*Abbreviations*: *FGR* fetal growth restriction, *LBW* low birth weight, *LGA* large for gestational age, *NICU* neonatal intensive care unit, *PE* preeclampsia, *RR* risk ratio, *SGA* small for gestational age

Outcomes of interest in our meta-analyses included pregnancy outcomes associated with glucose dysmetabolism according to the HAPO study, the published GDM core outcome set, and the outcomes reported in a previous systematic review [13, 17, 18, 21]. All studies that were extracted from a full-text article had detailed the growth reference standards for criteria of neonatal birth weight, but the studies from conference abstracts did not explain the reference standards used for their cohorts.

### Association between GRH and adverse pregnancy outcomes

**Fig. 2:**
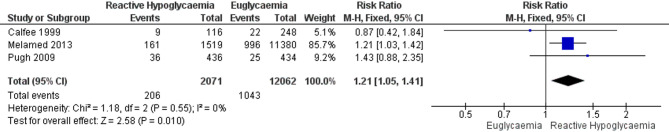
Forest plot of the pooled effect of estimate (RR) of FGR when GRH was compared with euglycaemia

Extracted data from included studies that reported adverse pregnancy outcomes were pooled to determine summary risk ratio (RR) for the associations between GRH and outcomes of interest (Table 3). Women with GRH were at a higher risk of delivering a baby that was SGA, LBW, and FGR (Fig. 2.) whilst they were at lower risk of delivering macrosomic/LGA infants as well as baby with hyperbilirubinaemia after birth. Neonates of women with GRH had a lower risk of delivered by caesarean section. This meta-analysis revealed no differences in the risk of other pregnancy outcomes related to glucose dysmetabolism (NICU admission, neonatal hypoglycaemia, low 5-minute APGAR score, and perinatal mortality).

**Table 3:** Key findings of the Meta-Analysis

Pregnancy Outcomes	Heterogeneity (I^2^)	Model	Pooled RR (95% CI)
SGA	59%	Random-effect	1.49 (1.33, 1.68)**
LGA	25%	Fixed-effect	0.74 (0.76, 0.82)**
LBW	67%	Random-effect	1.35 (1.13, 1.60)**
Macrosomia	0%	Fixed-effect	0.69 (0.61, 0.77)**
NICU admission	60%	Random-effect	1.23 (1.02, 1.49)*
APGAR score < 7	63%	Random-effect	1.88 (0.96, 3.68)
Caesarean section	76%	Random-effect	0.90 (0.79, 0.96)**
Shoulder dystocia	0%	Fixed-effect	0.50 (0.24, 1.05)
FGR	0%	Fixed-effect	1.21 (1.05, 1.41)*
Polyhydramnios	71%	Random-effect	0.94 (0.42, 2.12)
Neonatal hypoglycaemia	95%	Random-effect	1.54 (0.37, 6.44)
Neonatal hyperbilirubinaemia	0%	Fixed-effect	0.82 (0.72, 0.92)**
Perinatal mortality	12%	Fixed-effect	1.06 (0.73, 1.55)
Preterm delivery	89%	Random-effect	1.16 (0.88, 1.54)
PE	57%	Random-effect	0.90 (0.71, 1.16)
Postpartum hemorrhage	84%	Random-effect	0.59 (0.29,1.20)

*Abbreviations*: *FGR* fetal growth restriction, *LBW* low birth weight, *LGA* large for gestational age, *N/A* non-applicable, *NICU* neonatal intensive care unit, *PE* preeclampsia, *RR* risk ratio, *SGA* small for gestational age. Asterisk (*) symbol indicates significant value

*= *p*-value < 0.05; **=*p*-value < 0.01

### Subgroup analyses

**Fig. 3:**
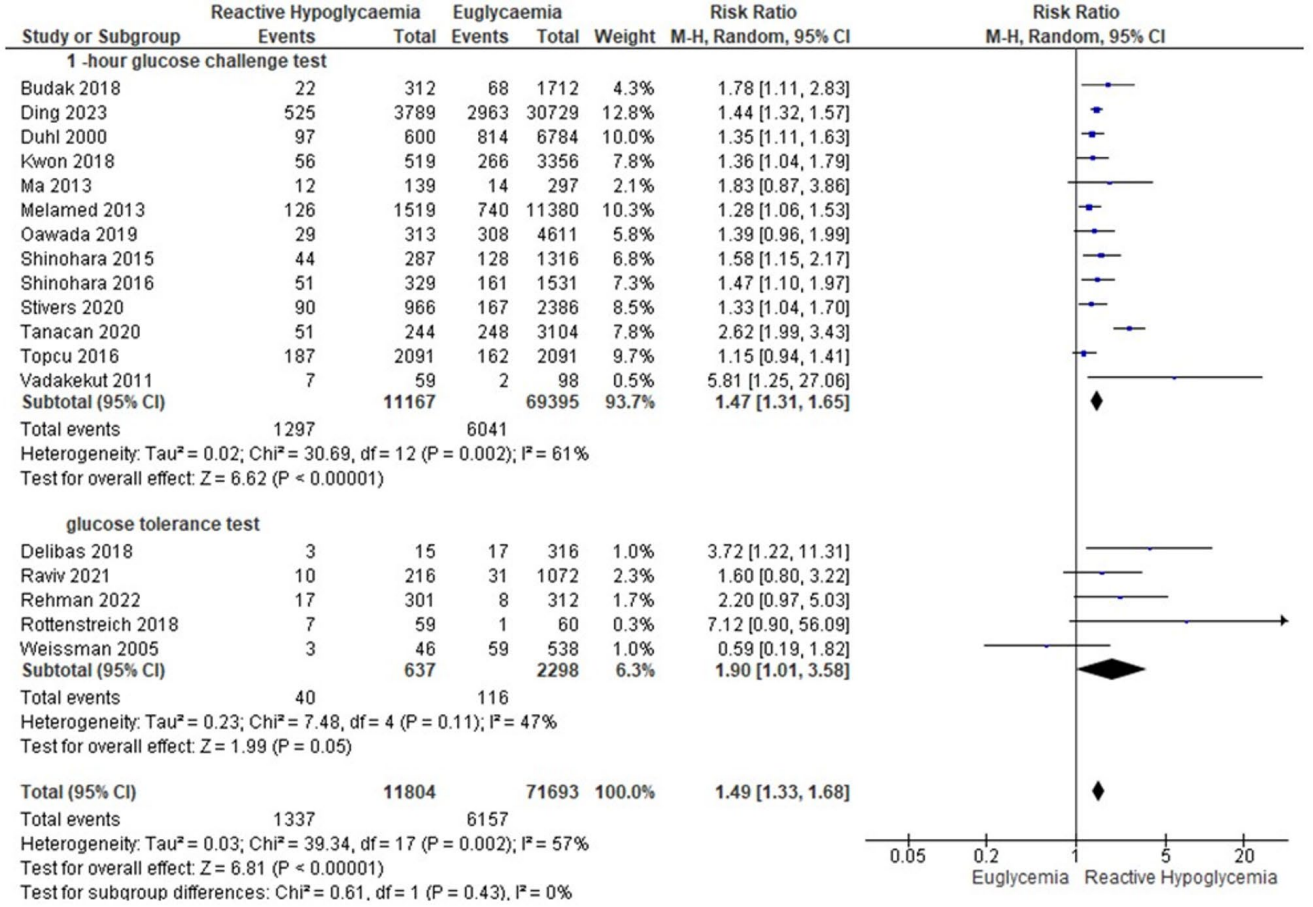
Forest plot of the subgroup analysis for GRH at 1-hour GCT and GTT to the risk of small for gestational age

**Fig. 4:**
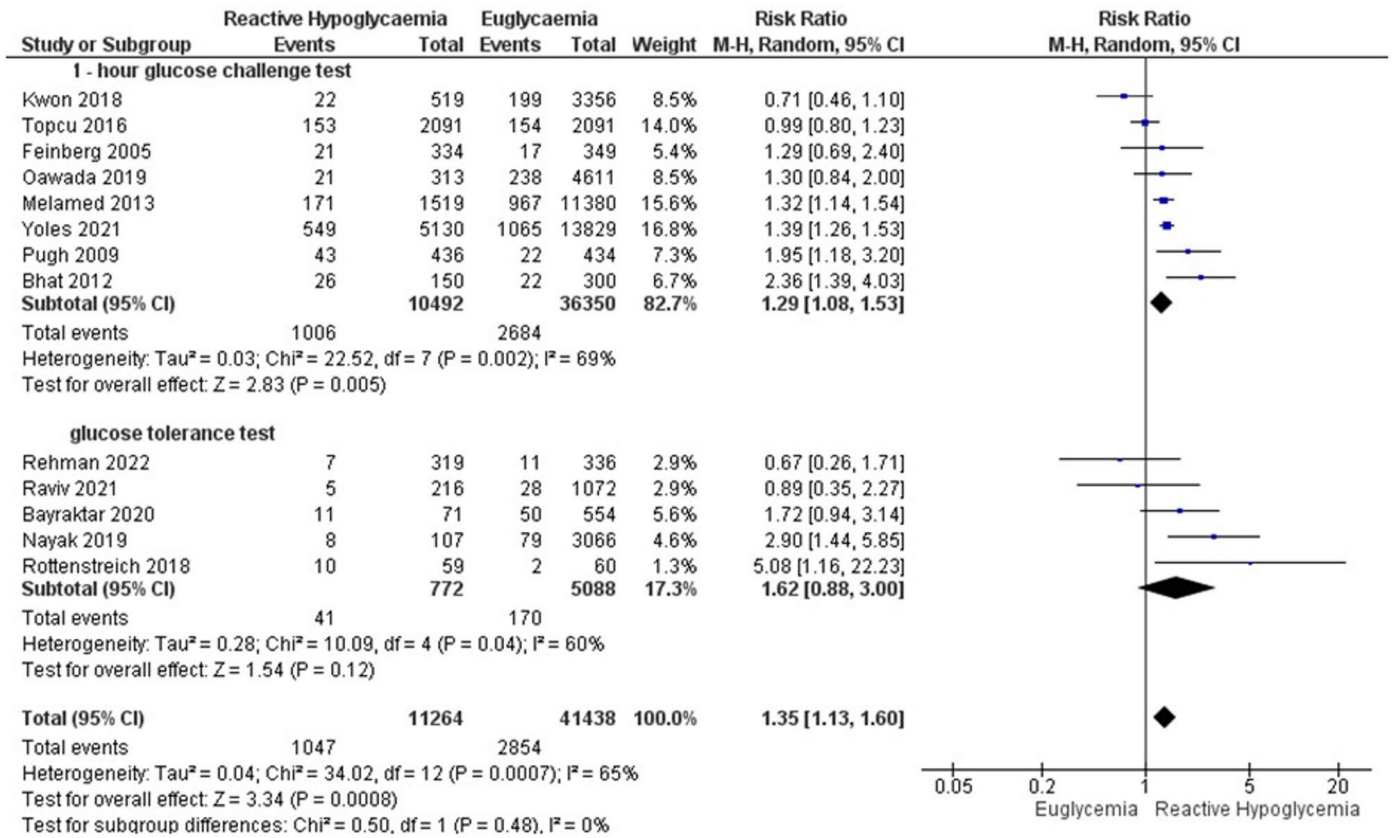
Forest plot of the subgroup analysis for GRH at 1-hour GCT and GTT to the risk of low birth weight

**Fig. 5:**
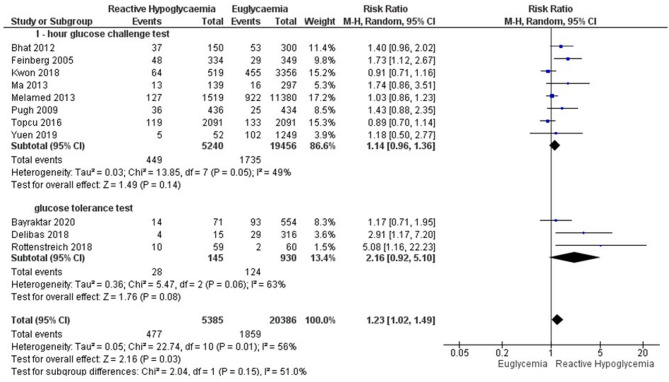
Forest plot of the subgroup analysis for GRH at 1-hour GCT and GTT to the risk of NICU admission

**Fig. 6:**
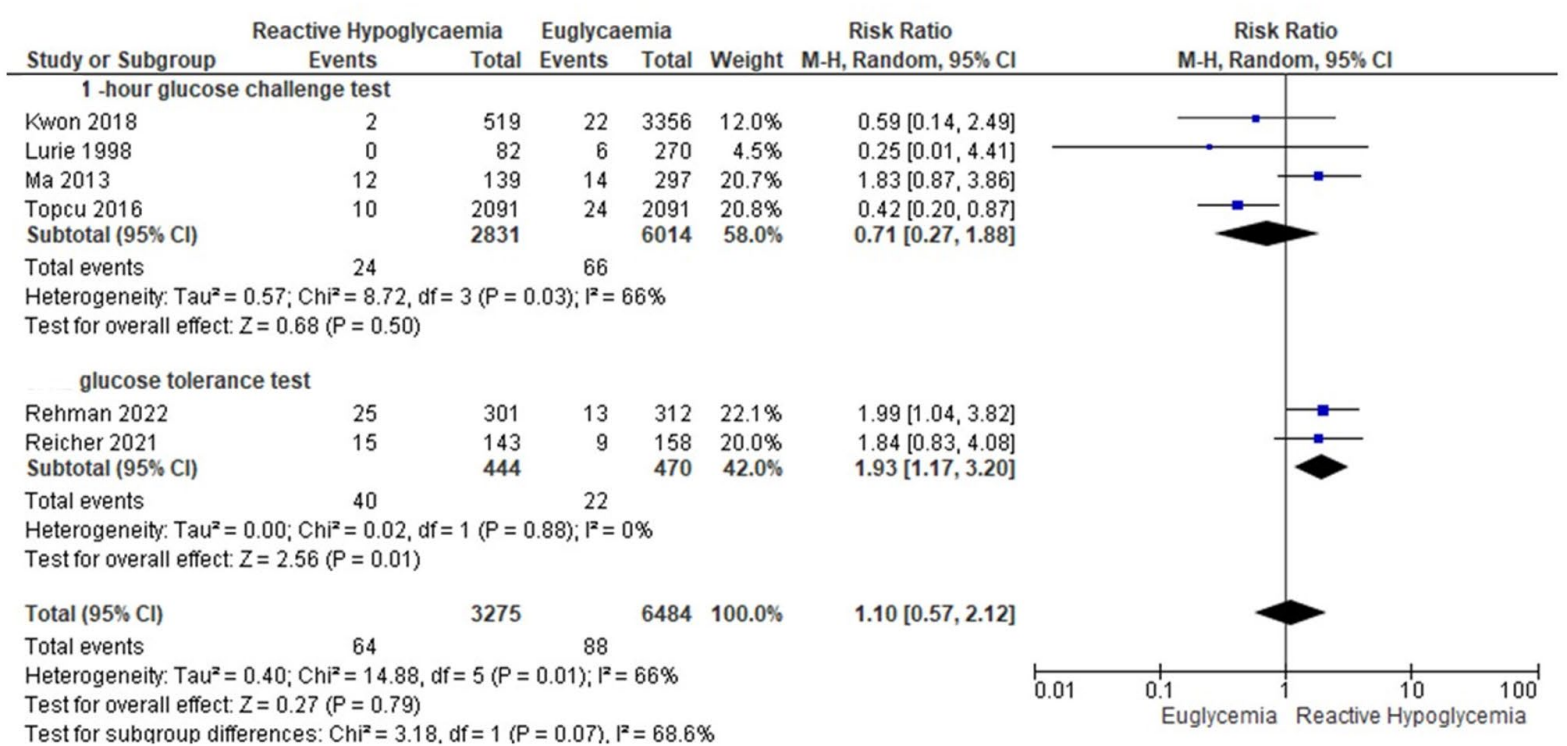
Forest plot of the subgroup analysis for GRH at 1-hour GCT and GTT to the risk of polyhydramnios

Subgroup analysis was based on a pragmatic classification of the glucose threshold value ((postload glucose < 5mmol/l, < 4 mmol/l, and < 3 mmol/l (Table 4.). Using a threshold for GRH diagnosis of glucose lower than 5 mmol/l, GRH was associated with an increased risk of SGA, LBW, FGR, and NICU admission. Using postload glucose < 4 mmol/l as a cutoff value for GRH, the risk of LBW still persisted. GRH using a threshold of postload glucose < 3 mmol/l was associated with an increased risk of NICU admission as well as LBW (Table 4.). GRH following 1-hour GCT was associated with increased risks of SGA (Fig. 3), LBW (Fig. 4), and FGR. GRH at GTT was associated with SGA, and polyhydramnios (Table 5; Figs. 5–6).

**Table 4:** Subgroup analysis based on plasma glucose threshold value

Pregnancy Outcomes	Postload glucose < 5 mmol/l	Postload glucose < 4 mmol/l	Postload glucose < 3 mmol/l
	Pooled RR (95% CI)	Pooled RR (95% CI)	Pooled RR (95% CI)
SGA	1.49 (1.33, 1.68)**	1.38 (1.09, 1.74)**	2.20 (0.49, 9.87)
LGA	0.74 (0.76, 0.82)**	0.83 (0.71, 0.97)*	0.57 (0.28, 1.18)
LBW	1.35 (1.13, 1.60)**	1.45 (1.22, 1.72)**	5.08 (1.16, 22.23)*
Macrosomia	0.69 (0.61, 0.77)**	0.80 (0.64, 1.01)	0.54 (0.25, 1.15)
NICU admission	1.23 (1.02, 1.49)*	1.40 (0.92, 2.12)	3.39 (1.56, 7.34)**
APGAR score < 7	1.88 (0.96, 3.68)	1.88 (0.96, 3.68)	N/A
Caesarean section	0.90 (0.79, 0.96)**	0.90 (0.79, 1.02)	0.80 (0.56, 1.15)
Shoulder dystocia	0.50 (0.24, 1.05)	0.50 (0.24, 1.05)	N/A
FGR	1.21 (1.05, 1.41)*	N/A	N/A
Polyhydramnios	0.94 (0.42, 2.12)	N/A	N/A
Neonatal hypoglycaemia	1.54 (0.37, 6.44)	7.10 (0.06, 823.09)	N/A
Neonatal hyperbilirubinaemia	0.82 (0.72, 0.92)**	N/A	N/A
Perinatal mortality	1.06 (0.73, 1.55)	2.46 (0.28, 21.28)	N/A
Preterm delivery	1.16 (0.88, 1.54)	1.17 (0.82, 1.68)	1.71 (0.38, 7.76)
PE	0.90 (0.71, 1.16)	0.93 (0.67, 1.30)	N/A
Postpartum hemorrhage	0.59 (0.29,1.20)	N/A	N/A

*Abbreviations*: *FGR* fetal growth restriction, *LBW* low birth weight, *LGA* large for gestational age, *N/A* not applicable, *NICU* neonatal intensive care unit, *PE* preeclampsia, *RR* risk ratio, *SGA* small for gestational age. Asterisk (*) symbol indicates significant value.

*= *p*-value < 0.05; **=*p*-value < 0.01

**Table 5:** Subgroup analysis based on glucose loading test (1-hour glucose challenge test vs. glucose tolerance test)

Pregnancy Outcomes	1-hour Glucose challenge test	Glucose tolerance test
	Pooled RR (95% CI)	Pooled RR (95% CI)
SGA	1.47 (1.31, 1.65)**	1.90 (1.01, 3.58)*
LGA	0.72 (0.65, 0.81)**	0.90 (0.69, 1.18)*
LBW	1.29 (1.08, 1.53)**	1.62 (0.88, 3.00)
Macrosomia	0.69 (0.62, 0.78)**	0.64 (0.43, 0.94)*
NICU admission	1.14 (0.96, 1.36)	2.16 (0.92, 5.10)
APGAR score < 7	1.46 (0.73, 2.89)	1.98 (0.47, 8.24)
Caesarean section	0.86 (0.77, 0.96)*	1.01 (0.83, 1.22)
Shoulder dystocia	0.11 (0.01, 1.77)	0.69 (0.32, 1.49)
FGR	1.21 (1.05, 1.41)*	N/A
Polyhydramnios	0.71 (0.27, 1.88)	1.93 (1.17, 3.20)**
Neonatal hypoglycaemia	1.64 (0.28, 9.74)	1.05 (0.36, 2.97)
Neonatal hyperbilirubinaemia	0.82 (0.72, 0.92)**	N/A
Perinatal mortality	1.04 (0.71, 1.52)	3.16 (0.13, 77.27)
Preterm delivery	1.10 (0.81, 1.50)	1,59 (0.70, 3.58)
PE	0.90 (0.71, 1.16)	0.99 (0.61, 1.61)
Postpartum hemorrhage	0.59 (0.29,1.20)	N/A

*Abbreviations*: *FGR* fetal growth restriction, *GCT* glucose challenge test, *GTT* glucose tolerance test, *LBW* low birth weight, *LGA* large for gestational age, *N/A* not applicable, *NICU* neonatal intensive care unit, *PE* preeclampsia, *PG* plasma glucose, *RR* risk ratio, *SGA* small for gestational age. Asterisk (*) symbol indicates significant value

*= *p*-value < 0.05; **=*p*-value < 0.01

## Discussion

In the meta-analysed literature, pregnant women with GRH value < 5 mmol/l had a high risk of having small babies, either diagnosed with SGA, LBW, or FGR while the risk of delivering LGA or macrosomia was reduced. Of outcomes associated with diabetes, only polyhydramnios was associated with GRH in this meta-analysis. Our meta-analysis agrees with the previous meta-analysis by Mitta [13]. Their meta-analysis showed an increased risk of SGA in women with a 1-hour GCT value < 5 mmol/l. However, they did not examine studies with GRH following GTT. Hypoglycaemia is typically defined in pregnant women with diabetes using a glucose level < 4 mmol/l in which neuroendocrine response is more observed and may need immediate treatment [47]. In a subgroup analysis, LBW still persisted with GRH threshold < 4 mmol/l. Published studies have used different cut-offs to define GRH. Physiological glucose in non-diabetic subjects may be lower, although precise limits are debated. Our meta-analysis suggests that in GRH with glucose value < 3 mmol/l (the most severe), there is an increased risk for baby to be admitted to NICU. Quansah et al. found that women with GDM and subsequent postpartum RH < 3.9 mmol/l have a better metabolic profile, with better insulin response than women with typical GDM and no postpartum RH [65]. Whereas it is possible that even severe GRH represents a milder and/or earlier form of undiagnosed glucose intolerance, there are no long-term studies of women with GRH alone without diagnosis of GDM. Women with GDM in pregnancy display a less sensitive maternal insulin response to a glycaemic stimulus compared to otherwise normal pregnancies [66]. GRH may reflect the first phase of glucose dysmetabolism before overt diabetes, a suboptimal primary insulin response to glucose loads, but this remains speculative. When the first-phase insulin response in insufficient, there may be a late and excessive second-phase insulin secretion, leading to reactive hypoglycaemia [14, 67]. In other words, GRH may be caused by a delay in the peak of first-phase insulin secretion in response to plasma glucose levels, followed by excessive secondary insulin response [68]; insulin response to glucose loads can also biphasic or triphasic [69]. The phenomenon may very well have multiple aetiologies. Altered gut transit or glucose absorption, abnormally increased insulin sensitivity, reduced insulin clearance, and changes in the hypothalamus-hypophysis axis also merit consideration. Maternal hypoglycaemia may decrease glucose availability to fetus [70] but that would not explain why GRH and FGR co-exist in women with insulin resistance or obesity or in women subsequently diagnosed with diabetes. Disorders in placental function may be key to untangling, at least in part, the pathophysiology leading to placental dysfunction and abnormal fetal growth [71, 72]. Although no studies have reported to date placental abnormalities in GRH, some reports have improved our understanding of how during pregnancy can affect the placenta [48]. Placental histopathology findings associated with diagnosed or suspected glucose dysmetabolism, including possibly GRH, include villous thrombosis and maturation disorders (fetal vascular malperfusion, delayed villous maturation), in turn associated with outcomes such as FGR and stillbirth [49, 56, 73–76].

Another very interesting and novel finding in the subgroup analysis of this study is the unusual combination of SGA with polyhydramnios in women with GRH after GTT. Although its incidence is low, the co-existence of polyhydramnios and SGA in pregnancies in the absence of any fetal congenital malformation has been reported previously [34, 55]. A study in two UK hospitals, published recently [61] found that GRH, defined as 2-hour GTT value lower than or equivalent to fasting level, was associated with polyhydramnios and mean birthweight similar to pregnancies complicated by diabetes, with babies overall heavier than controls. Outcomes typically related to diabetes such as abdominal circumference > 95th centile, induction of labour, perinatal infection, hypertensive disorders of pregnancy, neonatal hypoglycaemia, and ambiguous genitalia were also higher with GRH in preliminary analyses of data from one hospital alone [77] but this did not persist in the combined dataset. Some of the pregnancy complications that are relevant to diabetes are multifactorial and the associated pathophysiology is not always clearly understood [42, 78–80]. Other pre-existing maternal conditions such as obesity and dyslipidaemia often found in women with GDM may contribute to the occurrence of these outcomes and other complications typically related to pregnancy diabetes and hyperglycaemia [46, 81, 82]. 

Our study could have been limited by the fact that there is no agreed recommendation on the glucose value threshold and tests to diagnose women with GRH. The authors in the included studies applied different tests (GCT/GTT), criteria, and glucose value thresholds to diagnose GRH following glucose loading test, according to each study setting, resulting in considerable clinical heterogeneity. Moreover, Rottenstreich’s study included women with a history of post-bariatric surgery [57]. We acknowledge this clinical heterogeneity in the considered studies, including in adjustment for confounders in. To address the study heterogeneity, the meta-analysis was performed using a random effect model. Since there are no agreed thresholds to define GRH, our meta-analyses help elucidate the impact of different thresholds on outcomes, to inform future research. How and when GRH affects pregnancies remains to be determined, but this meta-analysis, including the subgroup analyses, shows that GRH is associated with adverse neonatal outcomes, regardless of the test or threshold used to diagnose GRH. Some analyses not included in PROSPERO protocol for this systematic review andmeta analysis were performed post-hoc at the request of the peer-reviewers.

Some of the pregnancy outcomes related to glucose dysmetabolism and/or insulin resistance were not reported well or at all in the published literature, so they could not be examined in the meta-analyses. Existing studies are also potentially severely limited by the possible inclusion of women with undiagnosed milder glucose dysmetabolism, sufficient to cause placental dysfunction but not to be diagnosed formally as diabetes, in the controls, particularly when less sensitive criteria are used. A well-powered study with well-defined controls is needed to elucidate differences in insulin secretory and function, mechanistic pathophysiology, and adverse outcomes in pregnancy with GRH, compared to both GDM and to controls with normal glucose tolerance more narrowly defined.

## Conclusions

This meta-analysis has shown that regardless of the glucose loading test used to define GRH, this typically undiagnosed maternal condition poses risks to pregnancy. The risks include small baby and polyhydramnios were higher in GRH at GTT. However, the combination of a small baby with polyhydramnios, seen with GRH, may confuse clinicians and preclude appropriate intervention.

Further studies will be key to determining the best cutoff value to diagnose GRH and intervene if needed. We must balance the need to eliminate preventable harm, with the imperative to avoid alarming women unnecessarily, at least until we know more about the underlying maternal metabolic associations. Whereas it might be too early to change clinical practice, it is clear that GRH in pregnancy should be studied further, including with regards to longer term outcomes in the offspring.

## Supplementary Information

Supplementary Material 1.

Supplementary Material 2.

Supplementary Material 3.

Supplementary Material 4.

Supplementary Material 5.

Supplementary Material 6.

## Data Availability

The datasets used and/or analysed during the current study are available from the corresponding author on reasonable request.

## Abbreviations

FGR: fetal growth restriction
HAPO: hyperglycaemia and adverse pregnancy outcomes
LBW: low birth weight
LGA: large for gestational age
N/A: not applicable
NICU: neonatal intensive care unit
PE: preeclampsia
PG: plasma glucose
GRH: reactive hypoglycaemia
RR: risk ratio
SGA: small for gestational age
