# Supplementary material for: Gestational reactive hypoglycaemia and adverse pregnancy outcomes: a systematic review and meta-analysis
Source: BMC Pregnancy Childbirth. 2025 Aug 26;25:888. doi: 10.1186/s12884-025-08016-x (PMC12379322; doi:10.1186/s12884-025-08016-x)
Supplement: Supplementary file 2 — Supplementary Material 2. [file 12884_2025_8016_MOESM2_ESM.pdf]

Centre for Reviews and Dissemination

Systematic review

A list of fields that can be edited in an update can be found here

1. * Review title.

Give the title of the review in English

Reactive hypoglycemia in pregnancy associated with adverse pregnancy outcomes related to glucose dysmetabolism: a systematic review and meta-analysis

2. Original language title.

For reviews in languages other than English, give the title in the original language. This will be displayed with the English language title.

3. * Anticipated or actual start date.

Give the date the systematic review started or is expected to start.

19/01/2023

4. * Anticipated completion date.

Give the date by which the review is expected to be completed.

01/08/2023

5. * Stage of review at time of this submission. [1 change]

This field uses answers to initial screening questions. It cannot be edited until after registration.

Tick the boxes to show which review tasks have been started and which have been completed.

Update this field each time any amendments are made to a published record.

The review has not yet started: No

Review stage	Started	Completed
Preliminary searches	Yes	Yes
Piloting of the study selection process	Yes	Yes
Formal screening of search results against eligibility criteria	Yes	Yes
Data extraction	Yes	Yes
Risk of bias (quality) assessment	Yes	Yes
Data analysis	Yes	Yes

Provide any other relevant information about the stage of the review here.

6. * Named contact.

The named contact is the guarantor for the accuracy of the information in the register record. This may be any member of the review team.

Muhammad Pradhiki Mahindra

Email salutation (e.g. "Dr Smith" or "Joanne") for correspondence:

Dr Mahindra

7. * Named contact email.

Give the electronic email address of the named contact.

rmjwmpm@ucl.ac.uk

8. Named contact address

Give the full institutional/organisational postal address for the named contact.

G15-Teaching Administration Office (2nd Floor), 86-96 Chenies Mews, London, WC1E 6HX, United Kingdom

9. Named contact phone number.

Give the telephone number for the named contact, including international dialling code.

07949039637

10. * Organisational affiliation of the review.

Full title of the organisational affiliations for this review and website address if available. This field may be

completed as 'None' if the review is not affiliated to any organisation.

University College London, EGA Institute for Women's Health

Organisation web address:

11. * Review team members and their organisational affiliations. [1. change]

Give the personal details and the organisational affiliations of each member of the review team. Affiliation refers to groups or organisations to which review team members belong. **NOTE: email and country now MUST be entered for each person, unless you are amending a published record.**

Dr Muhammad Mahindra. University College London, EGA Institute for Women's Health
Dr Michelle Jie. University College London Hospital
Dr Muhammad Pradhika Mapindra. University College London, EGA Institute for Women's Health
Ms Sana Rehman. University College London
Dr Debora Marletta. University College London
Dr Owen Vaughan. University College London, EGA Institute for Women's Health
Professor Anna David. University College London, EGA Institute for Women's Health
Dr Sara Hillman. University College London, EGA Institute for Women's Health
Professor Dimitrios Siassakos. University College London, EGA Institute for Women's Health

12. * Funding sources/sponsors.

Details of the individuals, organizations, groups, companies or other legal entities who have funded or sponsored the review.

Indonesian Endownment Fund for Education

Grant number(s)

State the funder, grant or award number and the date of award

13. * Conflicts of interest.

List actual or perceived conflicts of interest (financial or academic).

None

14. Collaborators.

Give the name and affiliation of any individuals or organisations who are working on the review but who are not listed as review team members. **NOTE: email and country must be completed for each person, unless you are amending a published record.**

15. * Review question.

State the review question(s) clearly and precisely. It may be appropriate to break very broad questions down into a series of related more specific questions. Questions may be framed or refined using PI(E)COS or similar where relevant.

Is reactive hypoglycemia in pregnancy associated with adverse pregnancy outcomes related to glucose dysmetabolism?

16. * Searches.

State the sources that will be searched (e.g. Medline). Give the search dates, and any restrictions (e.g. language or publication date). Do NOT enter the full search strategy (it may be provided as a link or attachment below.)

Search dates : 19 January 2023

Databases used : MEDLINE, Embase, Web of Science, Maternity and Infant Care Database (MIDIRS)

Publication date : any year up to 19 January 2023

Publication language : any language

17. URL to search strategy.

Upload a file with your search strategy, or an example of a search strategy for a specific database, (including the keywords) in pdf or word format. In doing so you are consenting to the file being made publicly accessible. Or provide a URL or link to the strategy. Do NOT provide links to your search **results**.

https://www.crd.york.ac.uk/PROSPEROFILES/355124_STRATEGY_20230121.pdf

Alternatively, upload your search strategy to CRD in pdf format. Please note that by doing so you are consenting to the file being made publicly accessible.

Do not make this file publicly available until the review is complete

18. * Condition or domain being studied.

Give a short description of the disease, condition or healthcare domain being studied in your systematic review.

Reactive hypoglycemia.

Abnormal fetal growth.

Diabetes-related pregnancy complications

19. * Participants/population.

Specify the participants or populations being studied in the review. The preferred format includes details of both inclusion and exclusion criteria.

Women with singleton pregnancy, not diagnosed with existing diabetes mellitus (type 1 and type 2 DM) or formal gestational diabetes mellitus

20. * Intervention(s), exposure(s).

Give full and clear descriptions or definitions of the interventions or the exposures to be reviewed. The preferred format includes details of both inclusion and exclusion criteria.

Reactive hypoglycaemia defined as low glucose values following glucose challenge test or/and 1, 2, 3 hour glucose tolerance test; as defined by authors

21. * Comparator(s)/control.

Where relevant, give details of the alternatives against which the intervention/exposure will be compared (e.g. another intervention or a non-exposed control group). The preferred format includes details of both inclusion and exclusion criteria.

Euglycemia defined as negative test for diabetes as defined by authors

22. * Types of study to be included.

Give details of the study designs (e.g. RCT) that are eligible for inclusion in the review. The preferred format includes both inclusion and exclusion criteria. If there are no restrictions on the types of study, this should be stated.

Observational studies, either cohort or case-control study

No restrictions

23. Context.

Give summary details of the setting or other relevant characteristics, which help define the inclusion or exclusion criteria.

Any definition of reactive hypoglycemia will be included.

24. * Main outcome(s).

Give the pre-specified main (most important) outcomes of the review, including details of how the outcome is defined and measured and when these measurement are made, if these are part of the review inclusion criteria.

GDM associated complications: cesarean delivery, 5-minute APGAR score 7, NICU admission, neonatal hypoglycemia, polyhydramnios, macrosomia, fetal growth restriction. shoulder dystocia, brachial plexus injury, stillbirth

Measures of effect

Please specify the effect measure(s) for you main outcome(s) e.g. relative risks, odds ratios, risk difference, and/or 'number needed to treat.

Relative risks and odds ratios of outcomes compared to euglycemia

25. * Additional outcome(s).

List the pre-specified additional outcomes of the review, with a similar level of detail to that required for main outcomes. Where there are no additional outcomes please state ‘None’ or ‘Not applicable’ as appropriate to the review

Other diabetes-related outcomes (neonatal hypothermia, ambiguous genitalia, perinatal infection)

Measures of effect

Please specify the effect measure(s) for you additional outcome(s) e.g. relative risks, odds ratios, risk difference, and/or 'number needed to treat.

Relative risks and odds ratios for binary outcomes, mean difference for continous outcomes

26. * Data extraction (selection and coding).

Describe how studies will be selected for inclusion. State what data will be extracted or obtained. State how this will be done and recorded.

Study Selection: Two reviewers independently performed article screening and full text study assessment for selected studies (MPM and MJ) according to eligibility criteria. Any disagreements would be done by a consensus and reported to the third person (DS). The article screening method was done using EndNote

Data extraction: Information that would be collected from included articles is the study design, methodology, participant demographics and characteristics, number of events with outcomes (SGA, LGA, neonatal birthweight, Low birth weight, and macrosomia) from hypoglycemia and nonhypoglycemia groups, according to each study definition. The corresponding authors would be emailed for unclear information or missing data. Any disagreements would be resolved by consensus with the third person (DS). Recording data and data extraction was done using excel spreadsheet.

27. * Risk of bias (quality) assessment.

State which characteristics of the studies will be assessed and/or any formal risk of bias/quality assessment tools that will be used.

The scale of Newcastle-Ottawa Scale for Cohort and Case-control Studies was used to assess internal validity. According to the scale, the characteristics that will be assessed are the representativeness of the exposed cohort, selection of non exposed cohort, ascertainment of exposure, demonstration of unpresent outcome at the beginning of study, comparability of cohorts based on study design or analysis, outcome assessment, adequacy of follow-up, and duration of the exposure to cause outcomes.

We are going to assess the quality for all selected studies for qualitative analysis. Studies with poor quality, based on the scoring system, would be excluded from quantitative meta analysis. Two reviewers performed this assessment and any disagreements would be resolved by consensus with the third and fourth person (DS and AD)

28. * Strategy for data synthesis.

Describe the methods you plan to use to synthesise data. This **must not be generic text** but should be **specific to your review** and describe how the proposed approach will be applied to your data. If meta-analysis is planned, describe the models to be used, methods to explore statistical heterogeneity, and software package to be used.

For the quantitative data analysis, the minimum number must be at least 2 studies with good and fair quality.

The data that will be synthesized are the analysed outcomes.

The effect of interest from each article would be extracted to specific excel spreadsheet (to normalize data into Mean and Standard deviation for continuous outcomes and combine the mean for several studies). Meta-analysis is going to be carried out using R statistical software database. Random effects will be considered due to different definition of hypoglycemia and clinical heterogeneity among the studies.

29. * Analysis of subgroups or subsets.

State any planned investigation of 'subgroups'. Be clear and specific about which type of study or participant will be included in each group or covariate investigated. State the planned analytic approach.

None

30. * Type and method of review.

Select the type of review, review method and health area from the lists below.

Type of review

Cost effectiveness

No

Diagnostic

No

Epidemiologic

No

Individual patient data (IPD) meta-analysis

No

Intervention

No

Living systematic review

No

Meta-analysis

Yes

Methodology

No

Narrative synthesis

No

Network meta-analysis
No

Pre-clinical
No

Prevention
No

Prognostic
No

Prospective meta-analysis (PMA)
No

Review of reviews
No

Service delivery
No

Synthesis of qualitative studies
No

Systematic review
Yes

Other
No

Health area of the review

Alcohol/substance misuse/abuse
No

Blood and immune system
No

Cancer
No

Cardiovascular
No

Care of the elderly
No

Child health
No

Complementary therapies

No

COVID-19
No

Crime and justice
No

Dental
No

Digestive system
No

Ear, nose and throat
No

Education
No

Endocrine and metabolic disorders
No

Eye disorders
No

General interest
No

Genetics
No

Health inequalities/health equity
No

Infections and infestations
No

International development
No

Mental health and behavioural conditions
No

Musculoskeletal
No

Neurological
No

Nursing
No

Obstetrics and gynaecology

Yes

Oral health

No

Palliative care

No

Perioperative care

No

Physiotherapy

No

Pregnancy and childbirth

Yes

Public health (including social determinants of health)

No

Rehabilitation

No

Respiratory disorders

No

Service delivery

No

Skin disorders

No

Social care

No

Surgery

No

Tropical Medicine

No

Urological

No

Wounds, injuries and accidents

No

Violence and abuse

No

31. Language.

Select each language individually to add it to the list below, use the bin icon to remove any added in error.

English

There is an English language summary.

32. * Country.

Select the country in which the review is being carried out. For multi-national collaborations select all the countries involved.

England

33. Other registration details.

Name any other organisation where the systematic review title or protocol is registered (e.g. Campbell, or The Joanna Briggs Institute) together with any unique identification number assigned by them. If extracted data will be stored and made available through a repository such as the Systematic Review Data Repository (SRDR), details and a link should be included here. If none, leave blank.

34. Reference and/or URL for published protocol. [1 change]

If the protocol for this review is published provide details (authors, title and journal details, preferably in Vancouver format)

Add web link to the published protocol.

https://www.crd.york.ac.uk/PROSPEROFILES/355124_PROTOCOL_20230829.pdf

Or, upload your published protocol here in pdf format. Note that the upload will be publicly accessible.

Yes I give permission for this file to be made publicly available

Please note that the information required in the PROSPERO registration form must be completed in full even if access to a protocol is given.

35. Dissemination plans.

Do you intend to publish the review on completion?

Yes

Give brief details of plans for communicating review findings.?

36. Keywords.

Give words or phrases that best describe the review. Separate keywords with a semicolon or new line. Keywords help PROSPERO users find your review (keywords do not appear in the public record but are included in searches). Be as specific and precise as possible. Avoid acronyms and abbreviations unless

these are in wide use.

Hypoglycaemia, Pregnancy Outcomes, Gestational Diabetes Mellitus, Glucose Screening Test

37. Details of any existing review of the same topic by the same authors.

If you are registering an update of an existing review give details of the earlier versions and include a full bibliographic reference, if available.

38. * Current review status. [1 change]

Update review status when the review is completed and when it is published.New registrations must be ongoing so this field is not editable for initial submission.

Please provide anticipated publication date

Review_Completed_not_published

39. Any additional information.

Provide any other information relevant to the registration of this review.

40. Details of final report/publication(s) or preprints if available. [1 change]

Leave empty until publication details are available OR you have a link to a preprint (NOTE: this field is not editable for initial submission). List authors, title and journal details preferably in Vancouver format.

Give the link to the published review or preprint.
