# Supplementary material for: Gestational reactive hypoglycaemia and adverse pregnancy outcomes: a systematic review and meta-analysis
Source: BMC Pregnancy Childbirth. 2025 Aug 26;25:888. doi: 10.1186/s12884-025-08016-x (PMC12379322; doi:10.1186/s12884-025-08016-x)
Supplement: Supplementary file 3 — Supplementary Material 3. [file 12884_2025_8016_MOESM3_ESM.docx]

**Table S1.** Search Strategy

| **MEDLINE search strategy <1946 to December 19, 2022>**   1. Hypoglycemia/ 2. [hypoglycemia.tw](http://hypoglycemia.tw). 3. [hypoglycaemia.tw](http://hypoglycaemia.tw). 4. low blood [glucose.tw](http://glucose.tw). 5. low GCT.tw. 6. exp Blood Glucose/an, me [Analysis, Metabolism] 7. glucose screen*.tw. 8. Blood Glucose/ or blood glucose*.tw. 9. or/1-8 10. Pregnancy Outcome/ or pregnancy outcome*.tw. 11. Pregnancy Complications/ or pregnancy complication*.tw. 12. low birth [weight.mp](http://weight.mp). or Infant, Low Birth Weight/ 13. Infant, Small for Gestational Age/ or small for gestational [age.tw](http://age.tw). 14. large for gestational [age.tw](http://age.tw). 15. Fetal Macrosomia/ or [macrosomia.tw](http://macrosomia.tw). 16. perinatal outcome*.tw. 17. 10 or 11 or 12 or 13 or 14 or 15 or 16 18. 9 and 17 19. exp animals/ not humans.sh. 20. 18 not 19 |
| --- |
| **Embase search strategy <1980 to 2022 Week 50>**   1. hypoglycemia/ 2. [hypoglycemia.tw](http://hypoglycemia.tw). 3. [hypoglycaemia.tw](http://hypoglycaemia.tw). 4. low blood [glucose.tw](http://glucose.tw). 5. low GCT.tw. 6. glucose blood level/ 7. glucose screen*.tw. 8. 1 or 2 or 3 or 4 or 5 or 6 or 7 9. pregnancy outcome/ 10. pregnancy outcome*.tw. 11. pregnancy complication/ 12. pregnancy complication*.tw. 13. low birth weight/ 14. low birth [weight.tw](http://weight.tw). 15. small for date infant/ 16. small for gestational [age.tw](http://age.tw). 17. large for gestational [age.tw](http://age.tw). 18. large for gestational age/ 19. macrosomia/ 20. [macrosomia.tw](http://macrosomia.tw). 21. perinatal outcome*.tw. 22. 9 or 10 or 11 or 12 or 13 or 14 or 15 or 16 or 17 or 18 or 19 or 20 or 21 23. 8 and 22 |

**Web of Science search strategy**

1. hypoglycemia OR hypoglycaemia OR low blood glucose OR low GCT OR glucose screen*
2. pregnancy outcome* OR pregnancy complication* OR low birth weight OR small for gestational age OR large for gestational age OR macrosomia OR perinatal outcome*
3. 1 AND 2

**Maternity & Infant Care Database (MIDIRS) search strategy <1971 to December 13, 2022>**

1. [hypoglycemia.tw](http://hypoglycemia.tw).
2. [hypoglycaemia.tw](http://hypoglycaemia.tw).
3. low blood [glucose.tw](http://glucose.tw).
4. low GCT.tw.
5. glucose screen*.tw.
6. pregnancy outcome*.tw.
7. pregnancy complication*.tw.
8. low birth [weight.tw](http://weight.tw).
9. small for gestational [age.tw](http://age.tw).
10. large for gestational [age.tw](http://age.tw).
11. [macrosomia.tw](http://macrosomia.tw).
12. perinatal outcome*.tw.
13. 1 or 2 or 3 or 4 or 5
14. 7 or 8 or 9 or 10 or 11 or 12
15. 13 and 14
