# Supplementary material for: Gestational reactive hypoglycaemia and adverse pregnancy outcomes: a systematic review and meta-analysis
Source: BMC Pregnancy Childbirth. 2025 Aug 26;25:888. doi: 10.1186/s12884-025-08016-x (PMC12379322; doi:10.1186/s12884-025-08016-x)
Supplement: Supplementary file 4 — Supplementary Material 4. [file 12884_2025_8016_MOESM4_ESM.docx]

| **Author, Year** | **Representativeness of the Exposed Cohort** | **Selection of the Non-Exposed Cohort** | **Ascertainment of Exposure** | **Outcome of Interest Was Not Present at Start of Study** | **Comparability of Cohorts on the Basis of the Design or Analysis** | **Assessment of Outcome** | **Follow-up Long Enough for Outcomes to Occur** | **Adequacy of Follow-up of Cohorts** | **Total Score** | | | |
| --- | --- | --- | --- | --- | --- | --- | --- | --- | --- | --- | --- | --- |
|  |  |  |  |  |  |  |  |  | **Selection Domain** | **Comparablity Domain** | **Outcome/ Exposure Domain** | **Quality** |
| Bhat, 2012 | * | * | no star | * | ** | * | * | * | 3 | 2 | 2 | Good |
| Bienstock, 2008 | no star | * | * | * | no star | * | * | * | 3 | 0 | 3 | Poor |
| Budak, 2018 | * | * | * | * | ** | * | * | * | 4 | 2 | 3 | Good |
| Calfee, 1999 | * | * | no star | * | ** | * | * | * | 3 | 2 | 3 | Good |
| Delibas, 2018 | * | * | * | * | ** | * | * | * | 4 | 2 | 3 | Good |
| Ding, 2023 | * | * | * | * | ** | * | * | * | 4 | 2 | 3 | Good |
| Duhl, 2000 | * | * | * | * | * | * | * | * | 4 | 1 | 3 | Good |
| Feinberg, 2005 | * | * | * | * | ** | * | * | * | 4 | 2 | 3 | Good |
| Kerenyi, 2009 | * | * | * | * | no star | * | * | no star | 4 | 0 | 2 | Poor |
| Kwon, 2015 | no star | no star | no star | * | * | no star | * | * | 1 | 1 | 2 | Poor |
| Kwon, 2018 | * | * | * | * | ** | * | * | * | 4 | 2 | 3 | Good |
| Lurie, 1998 **±** | no star | * | no star | * | * | * | * | * | 2 | 1 | 3 | Fair |
| Ma, 1998 | * | * | * | * | ** | * | * | * | 4 | 2 | 3 | Good |
| Melamed, 2013 | * | * | * | no star | ** | no star | * | * | 3 | 2 | 2 | Fair |
| Nayak, 2019 | * | * | * | * | * | * | * | no star | 4 | 1 | 2 | Good |
| Oawada, 2019 | * | * | * | * | ** | * | * | * | 4 | 2 | 3 | Good |
| Ong, 2008 | no star | * | * | * | * | * | * | * | 3 | 1 | 3 | Good |
| Pugh, 2009 | * | * | no star | * | * | * | * | * | 3 | 1 | 3 | Good |
| Raviv, 2021 | * | * | * | * | * | * | * | no star | 4 | 1 | 2 | Good |
| Rehman, 2022 | * | * | * | * | * | * | * | * | 4 | 1 | 3 | Good |
| Reicher, 2021 | * | * | * | * | * | * | * | * | 4 | 1 | 3 | Good |
| Rottenstreich, 2017 **±** | no star | * | * | * | * | * | * | * | 3 | 1 | 3 | Good |
| Scholl, 2021 | no star | * | * | * | no star | * | * | * | 3 | 0 | 3 | Poor |
| Shinohara, 2015 | * | * | * | * | * | * | * | no star | 4 | 1 | 2 | Good |
| Shinohara, 2016 | * | * | * | * | * | * | * | no star | 4 | 1 | 2 | Good |
| Stivers, 2020 | * | * | * | * | * | * | * | * | 4 | 1 | 3 | Good |
| Tanacan, 2020 | * | * | * | * | * | * | * | * | 4 | 1 | 3 | Good |
| TopÇu, 2016 | * | no star | * | * | * | * | * | no star | 3 | 1 | 2 | Good |
| Vadakekut, 2010 | * | * | * | * | * | * | * | * | 4 | 1 | 3 | Good |
| Vemareddy, 2009 | * | * | * | * | ** | * | * | * | 4 | 2 | 3 | Good |
| Yoles, 2021 | * | * | no star | * | * | no star | * | * | 3 | 1 | 2 | Good |
| Yuen, 2018 | * | no star | * | * | * | * | * | no star | 3 | 1 | 2 | Good |
| Weissman, 2005 | * | * | * | * | * | * | * | * | 4 | 1 | 3 | Good |
|  |  |  |  |  |  |  |  |  |  |  |  |  |
|  | **±** symbol > disagreement between reviewers, resolved by the senior author | | | |  |  |  |  |  |  |  |  |
